# Supplementary material for: Acyl chain asymmetry and polyunsaturation of brain phospholipids facilitate membrane vesiculation without leakage
Source: eLife. 2018 Mar 15;7:e34394. doi: 10.7554/eLife.34394 (PMC5903860; doi:10.7554/eLife.34394)
Supplement: Supplementary file 1. [file elife-34394-supp1.docx]

**Supplementary tables**

Manni et al. Acyl chain asymmetry and polyunsaturation of brain phospholipids facilitate membrane vesiculation without leakage

**Table S1.** Lipid composition of the LUVs used in most experiments and in all-atom simulations

| **Asymmetric** | **Fig 1 and Fig 1-sup fig1A**  **Fig 2B and Fig 2-sup fig 1**  **Fig 3 and Fig 3-sup fig 1B**  **Fig 4 and Fig 4-sup fig 1**  **Fig 6 sup fig 2** | **Symmetric** | **Fig 1 and Fig 1-sup fig 1A and B**  **Fig 2B** |
| --- | --- | --- | --- |
| **Lipids** | **mol %** | **Lipids** | **mol %** |
| C18:0-Cx PS | 30 | Cx-Cx PS | 30 |
| C18:0-Cx PE | 20 | Cx-Cx PE | 20 |
| C18:0-Cx PC | 16 | Cx-Cx PC | 16 |
| Cholesterol | 33 | Cholesterol | 33 |
| PIP_2_ | 1 | PIP_2_ | 1 |

**Asymmetric:** x = 18:1, 18:2, 20:4 and 22:6

**Symmetric:** x = 14:0, 18:1, 18:2, 20:4 and 22:6

**Table S2.** Lipid composition of the LUVs used for the NBD quenching assay

| **Asymmetric** | **Fig 2C** | **Symmetric** | **Fig 2C** |
| --- | --- | --- | --- |
| **Lipids** | **mol %** | **Lipids** | **mol %** |
| C18:0-Cx PS | 30 | Cx-Cx PS | 30 |
| C18:0-Cx PE | 19 | Cx-Cx PE | 19 |
| C18:0-Cx PC | 16 | Cx-Cx PC | 16 |
| Cholesterol | 33 | Cholesterol | 33 |
| PIP_2_ | 1 | PIP_2_ | 1 |
| C16:0-C16:0 PE-NBD | 1 | C16:0-C16:0 PE-NBD | 1 |

**Asymmetric:** x = 18:1, 18:2, 20:4 and 22:6

**Symmetric:** x = 14:0, 18:1, 18:2, 20:4 and 22:6

**Table S3**. Lipid composition of the LUVs used for the PIP_2_ dose response

|  | **Fig 3-sup fig 1A** | | | |
| --- | --- | --- | --- | --- |
| **Lipids** | **mol %** | **mol %** | **mol %** | **mol %** |
| C18:0-Cx PS | 30 | 30 | 30 | 30 |
| C18:0-Cx PE | 20 | 20 | 20 | 20 |
| C18:0-Cx PC | 17 | 16 | 14.5 | 12 |
| Cholesterol | 33 | 33 | 33 | 33 |
| PIP_2_ | 0 | 1 | 2.5 | 5 |

**Asymmetric:** x = 18:1, 18:2, 18:3, 20:4 and 22:6

**Table S4.** Lipid composition of the GUVs

| **Asymmetric** | **Fig 2D** | **Symmetric** | **Fig 2D** |
| --- | --- | --- | --- |
| **Lipids** | **mol %** | **Lipids** | **mol %** |
| Egg PC | 21 | Egg PC | 21 |
| C18:0-Cx PS | 19.9 | Cx-Cx PS | 19.9 |
| C18:0-Cx PE | 36 | Cx-Cx PE | 36 |
| Cholesterol | 20 | Cholesterol | 20 |
| PIP_2_ | 3 | PIP_2_ | 3 |
| Tx RED-DHPE | 0.1 | Tx RED-DHPE | 0.1 |

**Asymmetric:** x = 18:1, 20:4 and 22:6**Symmetric:** x = 22:6
